# Identifying temperature cues driving increased voltinism in a geometrid moth

**DOI:** 10.1007/s00442-025-05770-9

**Published:** 2025-07-09

**Authors:** Jemma Guthrie, Hannele M. Honkanen, Daniel T. Haydon, Colin E. Adams

**Affiliations:** 1 Scottish Centre for Ecology and the Natural Environment, School of Biodiversity, One Health and Veterinary Medicine, University of Glasgow, Glasgow, UK; 2 School of Biodiversity, One Health and Veterinary Medicine, University of Glasgow, Glasgow, UK

**Keywords:** Lepidoptera, Development, Climate change, Long-term data, Population dynamics

## Abstract

Identifying the environmental cues that determine the timing of developmental processes is vital to understanding the effects of climate change on populations. However, as developmental processes are inherently difficult to measure directly at the population level, the drivers and potential consequences of change in their timings remain unknown in most species. Here we explore the use of long-term monitoring data for assessments of change in the number of generations per year and its impact on abundance, demonstrating new applications for a rapidly growing data source. Data derived from a light trap in west-central Scotland operated over 56 years (1968 to 2023) showed that the small phoenix moth, *Ecliptopera silaceata,* switched from a univoltine to bivoltine generation pattern. This voltinism change was predicted by an increased minimum temperature in a critical time window towards the later part of the first generation’s flight period. The population shows positive density dependence and the change in voltinism has no significant negative effect on population size, indicating no evidence of a developmental trap that has been postulated for other species. These results identify some of the proximate mechanisms of developmental responses to climate change in general and in voltinism in particular, specifically highlighting the importance of sustained temperature above minimum thresholds for development. These results could also help to make predictions about future changes in population sizes under climate change and increasing voltinism, in addition to how these changes may differ between species.

## Introduction

The environment is an important driver of developmental rate in ectotherms. Development in Lepidoptera, as with ectotherms more widely, is thought to be controlled in large part by temperature (Brown et al. 2004; Dixon et al. 2009; Régnière et al. 2012), in combination with the seasonal cues provided by photoperiod (Gotthard et al. 2000; McGregor 1997). A common developmental pattern in ectotherms is for higher temperatures to facilitate continuous development within a temporal window defined by photoperiod, but for temperature to cease being the main driver outside the boundaries of this photoperiod-defined window (Grevstad & Coop 2015; Koštál, 2006). This pattern of combined temperature- and photoperiod- controlled development has the potential for developmental conflict if these cues mismatch, as is possible with climate change or during range expansion. Environmental temperature can affect key developmental processes such as growth rate (Barton et al. 2014) and timing of transitions between life stages (e.g. hatching or pupation) (Belitz et al. 2021), which can alter the time between generations.

Voltinism, the number of generations an insect has each year (Kogan & Prokopy 2009), is controlled by development pace, and can be influenced by environmental conditions to which individuals are exposed. Multivoltinism, or the occurrence of more than one full life cycle of an animal within a single year, is only possible where there is a window of environmental conditions suitable for development to adult life stages of a duration greater than that of the period of juvenile development and adult lifespan of one generation. As temperatures increase with climate change, the time window of suitable temperatures for development in temperate regions is likely to expand, creating the opportunity for species with facultative multivoltinism to produce more generations each year (Altermatt 2010; Pöyry et al. 2011).

Changes in development leading to changes in voltinism can have consequences at a population level, with important ecological implications. Facultatively multivoltine species have the potential to benefit from increasing temperatures, with increased numbers of generations per year providing opportunities for accelerated population growth, whilst obligate univoltine species will not experience this benefit (Macgregor et al. 2019). However, increased voltinism could also have deleterious effects. Additional generations may emerge later in the year during periods of suitable conditions for development, but these suitable conditions may not continue throughout subsequent life stages. This may lead to potential negative survival consequences for the overwintering component of the population, thus leading to what has been described as a developmental trap (Kerr et al. 2019). Consequently, voltinism and how it manifests in response to changing conditions could alter abundance of invertebrate populations and the composition of invertebrate communities, with important effects for the wider ecosystem.

In species for which developmental responses to thermal and photoperiodic cues are well understood, demographical models can be used to predict changes in voltinism in response to changing environmental conditions and how these changes may affect the population. Chen et al. (2011) predicted a mean increase in voltinism of 0.3–0.8 generations per year in the grape berry moth (*Paralobesia viteana* (Clemens, 1860)) in response to projected temperature increases across a range of climate change scenarios (Chen et al. 2011). Similar models for the mustard white butterfly (*Pieris oleracea* (Harris, 1829)) predicted increased voltinism across its range in the USA, with this facilitating higher population growth rates in southern parts of its range. However, increased voltinism was predicted to result in a developmental trap for final generations in each year further north. This is because the window of suitable conditions for development would not extend throughout the lifespan of the latest generation of each year (Kerr et al. 2019). Evidence of similar trends has also been observed in wild populations. Choi et al. (2011) provide an example of a likely temperature-driven increase in voltinism with potential negative consequences for population fitness in the pine moth (*Dendrolimus spectabilis* (Butler, 1877)) in South Korea. This moth has shifted from univoltinism to bivoltinism, and laboratory studies have shown this is a response to temperature increase. Furthermore, individuals of the second generation in bivoltine populations were smaller and had lower fecundity. This reduced fitness may be due to adverse conditions in the emergence period of the second generation, perhaps due to lower food availability and less favourable temperatures (Choi et al. 2011).

To predict how ectothermic animals with variable voltinism will respond to changing environmental conditions, it is vital to determine the points in their life history which are the most important drivers of development, and the thresholds of environmental conditions which can drive changes in development. Few species have been studied in sufficient depth to detect the point in the life cycle at which environmental conditions determine whether the subsequent generation will develop immediately or enter diapause, with most research carried out on agricultural pests (Tobin et al. 2001, 2003).

Data from continuous monitoring by light trapping can provide records of flight periods of Lepidoptera (and some other groups). Light trapping data and weather data collected over sufficiently long timescales for changes in climate to be detected can be used to test the effects of environmental conditions on voltinism. In particular, such data may help identify the occurrence of critical time windows in which environmental conditions may particularly strongly influence developmental rate. These data can also be used to determine how changes in voltinism affect abundance.

Many species show variation in voltinism across their range, particularly as the length of the growing season varies with latitude (Kong et al. 2019). The small phoenix moth (*Ecliptopera silaceata* (Denis & Schiffermüller, 1775)) shows variation in voltinism across its geographical range. This moth lives in a wide range of habitats and the larvae feed primarily on willowherbs (*Epilobium spp.*) (Chinery 2005). *E. silaceata* typically shows bivoltinism in the southern part of its range and univoltinism further north. The first generation of this species flies from May to July, with the second generation flying in August and September where it occurs, before overwintering as pupae (Waring & Townsend 2018).

### Aims & hypotheses

In this study, we use long-term data from a light trap operated to collect moths at one site in west-central Scotland at which *E. silaceata* has been commonly recorded. We use these data to test whether the number of flight periods per year has changed in this population over 56 years. We also investigate whether any change in number of flight periods can be attributed to changes in environmental conditions, and whether this may have population-level consequences. We test three hypotheses:

Hypothesis One: the number of flight periods per year in this population has increased over time

Hypothesis Two: increased environmental temperature within a particular annual window over the time series can explain the occurrence of a second flight period

Hypothesis Three: the occurrence of a second flight period has facilitated population growth.

## Methods

### Sampling methods

A Rothamsted light trap (Williams 1948) has been operating at the Scottish Centre for Ecology and the Natural Environment (SCENE), at Rowardennan (east Loch Lomondside) in west-central Scotland (56.128877, -4.611735), continuously since 1968 as part of the Rothamsted Insect Survey. To date this trap has generated a 57-year data set of moth catches, collated and identified by the Rothamsted Insect Survey (RIS 2021). The trap uses a 200W tungsten filament lightbulb to attract insects including moths into a clear funnel, to which is attached a collection jar. A peristaltic pump is attached to the collection jar and programmed to pump a small dose of tetrachloroethylene into the jar during the night, which kills the specimens in the jar. Samples are collected from the light trap daily and identified to species level where possible. Due to equipment failures, gaps in the data set occur in 1973, 1993 and 2017, meaning these years could not be included in analysis.

### Analysis methods

All analyses were conducted in R (R Core Team 2021), with figures created using *ggplot2* (Wickham 2016). The data used in this study spanned 56 years from 1968 to 2023. In addition to the Rothamsted Insect Survey data, weather data were obtained from the Met Office HadUK-Grid data set (Hollis et al. 2018). Daily maximum and minimum temperatures were extracted at a 1km^2^ spatial resolution at the light trap site (OS grid reference NS3795) for the duration of the moth data set. These data were also used to estimate daily mean temperature.

A combination of methods was used to estimate the number of flight periods in each year. Initial inspection of the data suggested that catches of *E. silaceata* could be grouped into two clusters, one occurring approximately from May to July, and the second in August and September. An approach used by Wepprich et al. (2025) in categorising voltinism using Gaussian mixture models was adapted to determine in which years the data supported the presence of two flight periods. Catches of *E. silaceata* were grouped by week. The frequency of *E. silaceata* catches in each week of the year was modelled using a Gaussian mixture model with the package *mixtools* (Benaglia et al. 2009). This model was used to determine the level of support for grouping the data, across all years, into two distributions. The model also provided posterior probabilities of each data point belonging to each distribution, which were used to determine whether individual years showed one or two flight periods (similar to Wepprich et al. (2025)). Years containing at least one data point with a posterior probability greater than 0.999 of belonging to the second Gaussian distribution were labelled as years with two flight periods, whilst all other years were labelled as having one flight period. This conservative approach was taken due to the possibility of long tails on the distributions, to avoid late-flying individuals from a first flight period being mistakenly attributed to a second flight period. In addition to the years when equipment failure led to no individuals of *E. silaceata* being captured, there were three additional years with no catches of this species (1972, 1996 & 1997), meaning these years were not included in this analysis. In 1 year (2019), no *E. silaceata* individuals were captured during the first flight period, but were captured in the second flight period, and this year was classed as having two flight periods. Presence of a second flight period within each year was coded as binomial data, with 0 representing only one flight period and 1 showing occurrence of a second flight period.

To test whether the number of flight periods per year changed throughout the dataset, a generalised linear model (GLM) with a binomial distribution was used, with presence of a second flight period as the response variable and year as a continuous explanatory variable. Results of binomial GLMs were interpreted using odds ratios calculated with the *questionr* package (Barnier et al. 2025).

Sliding time window regressions (Phillimore et al. 2012; Roberts et al. 2015; Simmonds et al. 2019) were used to identify the time period in which each of maximum, minimum and mean daily temperature had the strongest effect on the presence of a second flight period. For each temperature variable, mean values for each year were calculated across windows of durations varying from 5 to 60 days. Windows of each duration were calculated with the start date varying by one day, ranging from the 23rd of September (22nd September in leap years) to the 31st of July of the following year (30th July in leap years). This interval was chosen to range from the latest date at which the potential second flight period was observed (represented by the latest date of observation throughout the dataset: 23rd September) and the earliest date (31st July) at which the second flight period is thought to occur based on posterior probabilities from the Gaussian mixture model, which only exceed 0.999 after the 30th week of the year, which ends on the 30th July. These sliding time window temperature variables were constructed using the *RcppRoll* package (Ushey 2018). For each temperature variable, binomial GLMs modelling the presence of a second flight period in each year on the mean value within each window were carried out. Pseudo-R^2^ was calculated for each GLM using the *pscl* package (Zeileis et al. 2008) to determine during which time window each temperature variable best explained presence of a second flight period. The best model for each temperature variable was compared using pseudo-R^2^ to determine whether a critical window for maximum, minimum or mean temperature best explained presence of a second flight period.

The final chosen models were examined in several ways, following methods outlined by Simmonds et al. (2019). Only the temperature variable (i.e. minimum, mean or maximum temperature) which yielded the critical window with the highest pseudo-R^2^ was scrutinised. We first examined the effect of the duration and timing of the data set used in the sliding time window regression on the output of the final model to test for potential changes in reaction norms across the data set. This was performed by dividing the 50-year data set used in the sliding window regression into 10 subsets by removing decades from the data set sequentially, both from the beginning and the end, decreasing from the full 50 years of data down to 10-year subsets. Sliding window regression was carried out on each of these subsets of data following the method described above for the full data set. For each subset, a final model with highest pseudo-R^2^ was chosen, from which the effect size, midpoint of the corresponding temperature time window, and duration of the temperature time window were extracted. Linear models were used to test the effect of timing and duration of the subset of data used to build the model (the year in which the subset commenced, and the number of years included in the subset) on each of the three outputs (effect size, midpoint of critical time window and duration of critical time window).

The predictive performance of the sliding window regression method was quantified using K-fold cross validation, again following Simmonds et al. (2019). Ten subsets of data were constructed by removing 5-year blocks from the data in turn. Each of these subsets was used in sliding window regression following the methods described above. The best model found for each data subset was used to predict the presence or absence of a second flight period in each of the 5 years of data not included in the subset used to construct the model. The predictions were coded as binomial data, with a predicted probability of occurrence of a second flight period greater than 0.5 classed as 1, whilst a probability less than 0.5 was classed as 0. Bias in these predictions was assessed by calculating the raw discrepancy between the predictions and the observed data (the sum of the differences between the two), with a positive or negative sign indicating prediction bias. The accuracy of the predictions from the models was calculated by the total number of predictions which matched the observed data divided by the total number of predictions.

Following validation of the final model, the temperature threshold in the critical window at which the probability of occurrence of a second flight period first exceeded 0.5 was determined.

A linear model was used to test whether temperature in the critical window for occurrence of a second flight period changed over the time series. The mean value of the temperature variable in the window which best explained presence of a second flight period was modelled as a normally distributed response variable, which was confirmed from visual examination of model residuals, whilst year was included as a continuous explanatory variable.

Effects of the presence of a second flight period on abundance were tested using generalised linear models with negative binomial error distribution using the *glmmTMB* package (Brooks et al. 2017), with number of individuals captured during the first flight period of each year as the response variable. Years in which equipment failure led to no catches of *E. silaceata* (1973, 1993 & 2017) were excluded from the data, but years with no recorded equipment failure in which no *E. silaceata* were caught either overall (1972, 1996 & 1997) or in the first flight period (2019) were included in the analysis as zero counts. The year following each excluded year was also removed from the data, enabling temporal lags in abundance to be used as an explanatory variable. To account for effects of temperature on abundance, which may manifest in parts of the life cycle prior to emergence as adults, mean temperature was calculated between October of the previous year and June of each year, to include most of the life cycle of the first flight period in each year. The most complex model explaining abundance of the first flight period included annual abundance during the previous flight period (the second flight period from the previous year following a year with two flight periods, or the only flight period in the previous year following a year with one flight period) as a continuous explanatory variable, the number of flight periods from the previous year as a categorical explanatory variable with two levels, an interaction of these variables, and mean temperature from October to June. A top-down model selection approach was used by removing explanatory variables and comparing models using likelihood ratio tests with the *lmtest* package (Zeileis & Hothorn 2002) to test the significance of each fixed effect. Diagnostic analyses were conducted using the *DHARMa* package (Hartig 2022) to confirm that model assumptions were met.

## Results

**Fig. 1:**
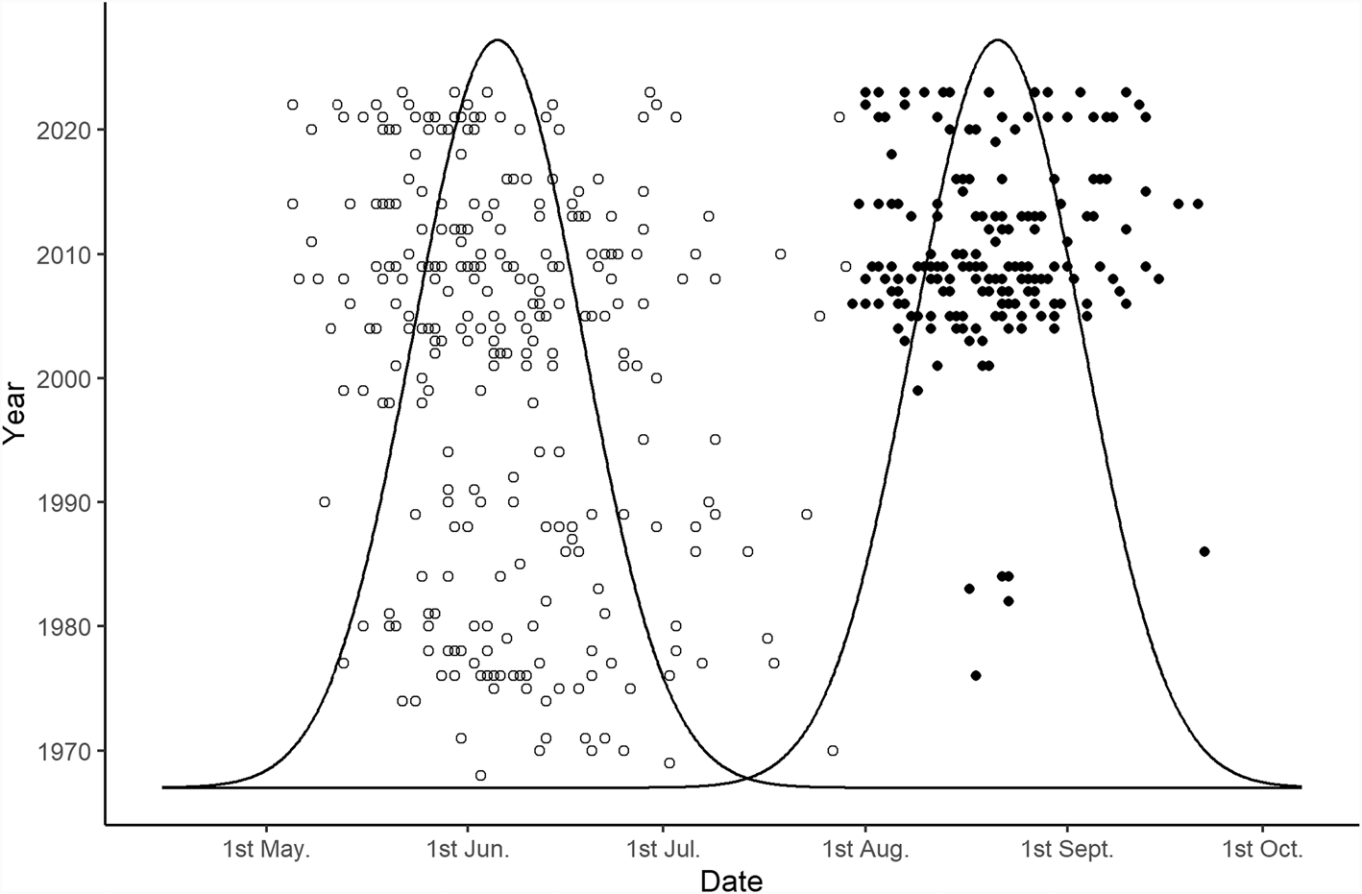
Plot showing the two Gaussian distributions describing the catch dates of *E. silaceata* generated by the Gaussian mixture model. Points show observed catch dates of *E. silaceata* (n = 776) ordered by year on the Y axis, with open points showing those attributed to a first annual flight period and filled points belonging to a second annual flight period

Preliminary examination of the data suggested that *E. silaceata* generally showed one flight period per year in early parts of the time series, before changing consistently to two flight periods in later years. A Gaussian mixture model of catches grouped by week suggested that the data came from two distributions, the first with a mean occurring at 22.4 weeks (late May/early June), and the second with a mean at 33.3 weeks (mid-August) (a common standard deviation of 1.9 weeks was estimated for both distributions) (Fig. 1). Using posterior probabilities generated by this model for each data point coming from either distribution, 28 years contained data points with a probability > 0.999 of coming from the second distribution. From 2003 onwards, there was a second flight period every year, whilst this occurred much more sporadically from 1968–2002, occurring in only 7 of the initial 34 years of the data set.

**Fig. 2:**
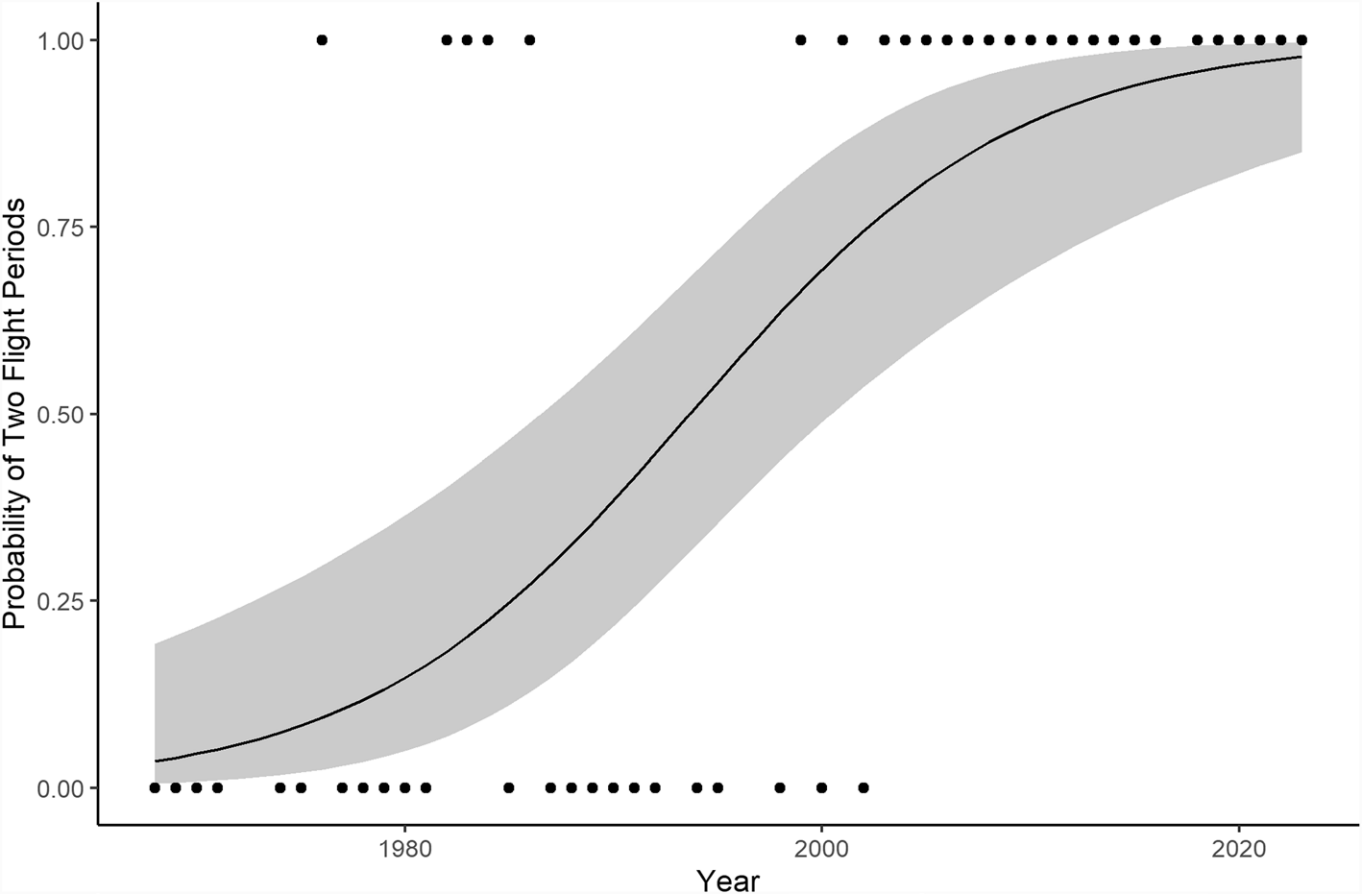
Plot of a binomial GLM of the presence of two flight periods per year in *E. silaceata* on year. Points show observed data, where 0 represents one flight period and 1 represents two flight periods (*n* = 50). Solid line shows the fitted curve from binomial GLM. The shaded area shows the 95% confidence interval

A binomial GLM of presence of a second flight period against year found that year had a significant positive effect on the presence of a second flight period (*p* < 0.001; Fig. 2). The odds ratio generated from the effect of year on number of flight periods per year was 1.14 (95% CI 1.07–1.23), meaning that with every increasing year, the odds of occurrence of a second flight period was 1.14 times greater.

Binomial GLMs using sliding time windows of minimum, maximum and mean daily temperature in relation to number of flight periods per year found that minimum temperature in a critical time window best explained presence of a second flight period (Table 1). The critical window for minimum temperature was a 49-day window from the 1st of June to 19th of July and had the highest pseudo-R^2^ of any model (0.406).

**Table 1:** Model parameters (temperature variable, window duration, window start date and window end date), odds ratio, 95% confidence interval and pseudo-R^2^ of best binomial GLMs of presence of a second flight period explained by each temperature variable (maximum, minimum and mean temperature)

Temperature variable	Window duration (days)	Window start date	Window end date	Odds ratio [95% CI]	Pseudo-R^2^
Maximum	60	30th May	28th July	3.86 [1.89, 9.78]	0.288
Minimum	49	1st June	19th July	16.24 [4.53, 92.47]	0.406
Mean	57	31st May	26th July	8.00 [2.92, 31.00]	0.354

We found no significant change in the midpoint of the critical time window chosen by sliding window regressions conducted using data subsets varying in timing and duration (*p* > 0.05). However, the duration of the critical window was significantly smaller when generated from more recent data, decreasing by 4.19 days for every increasing decade in start date of the data subset (t_1,12_ = -3.0, *p* < 0.05), although there was no significant effect of the duration of the data subset on the duration of the critical window (*p* > 0.05). The effect of temperature in the critical window on presence of a second flight period was unaffected by duration of the data subset (*p* > 0.05), but was marginally reduced by later start dates of the subset, reducing the odds by 3.34 for every increasing decade in the start of the data subset (*p* = 0.085). K-fold cross validation showed 72% accuracy in predictive performance of models across data subsets and showed no bias in predictions (raw discrepancy = 0).

**Fig. 3:**
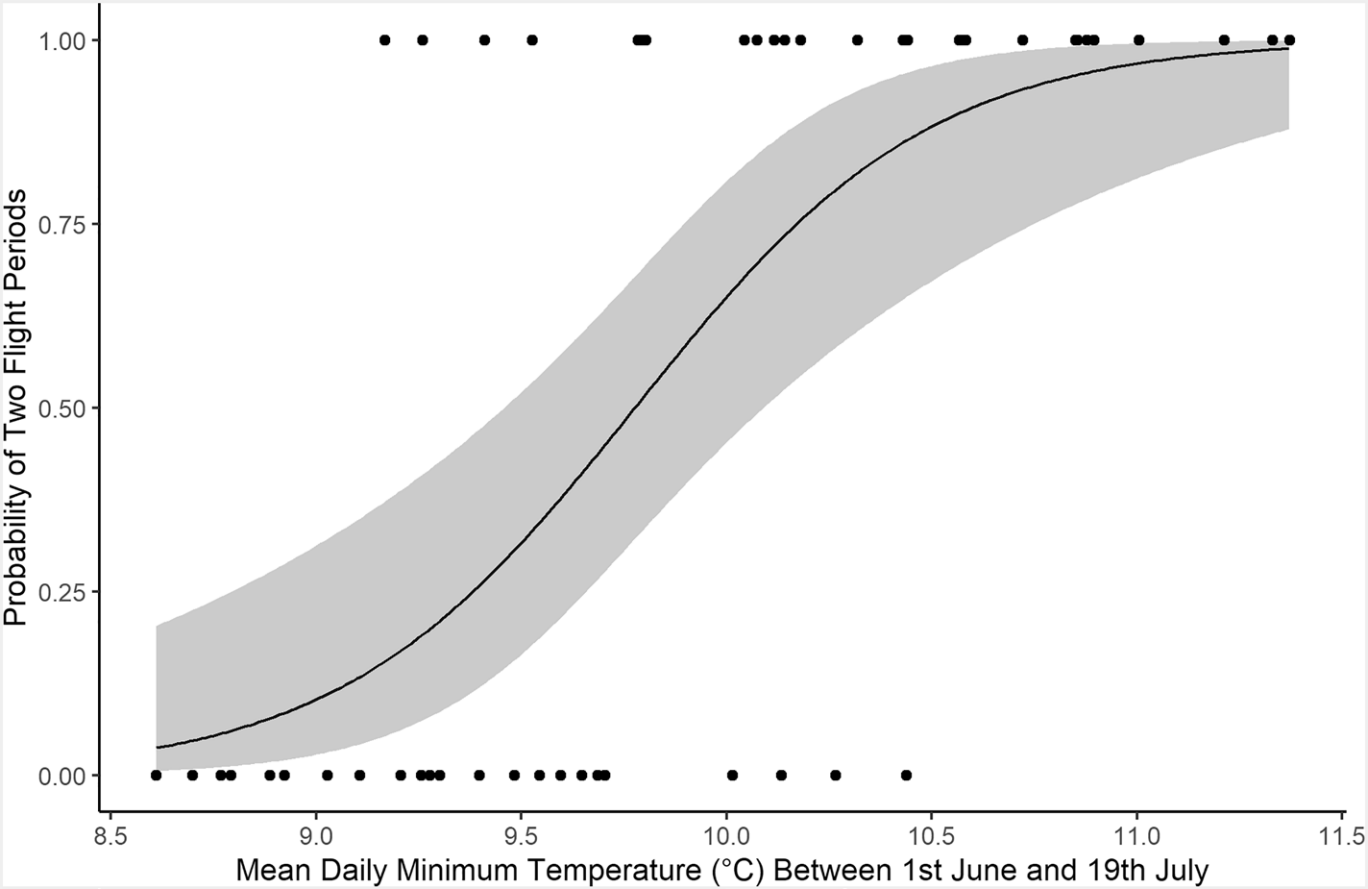
Plot of binomial GLM of the presence of a second flight period on the most informative critical temperature window (mean daily minimum temperature between 1st June and 19th July). Points show observed data (*n* = 50). Solid line shows fitted curve from binomial GLM. The shaded area shows the 95% confidence interval

The effect size (as measured by the odds ratio of presence of a second flight period per degree increase in temperature) was 16.24 (95% CI 4.53–92.47) (Fig. 3). Using this model, the mean daily minimum temperature during this window at which the probability of a second flight period first exceeds 0.5 occurs at 10.0 °C.

**Fig. 4:**
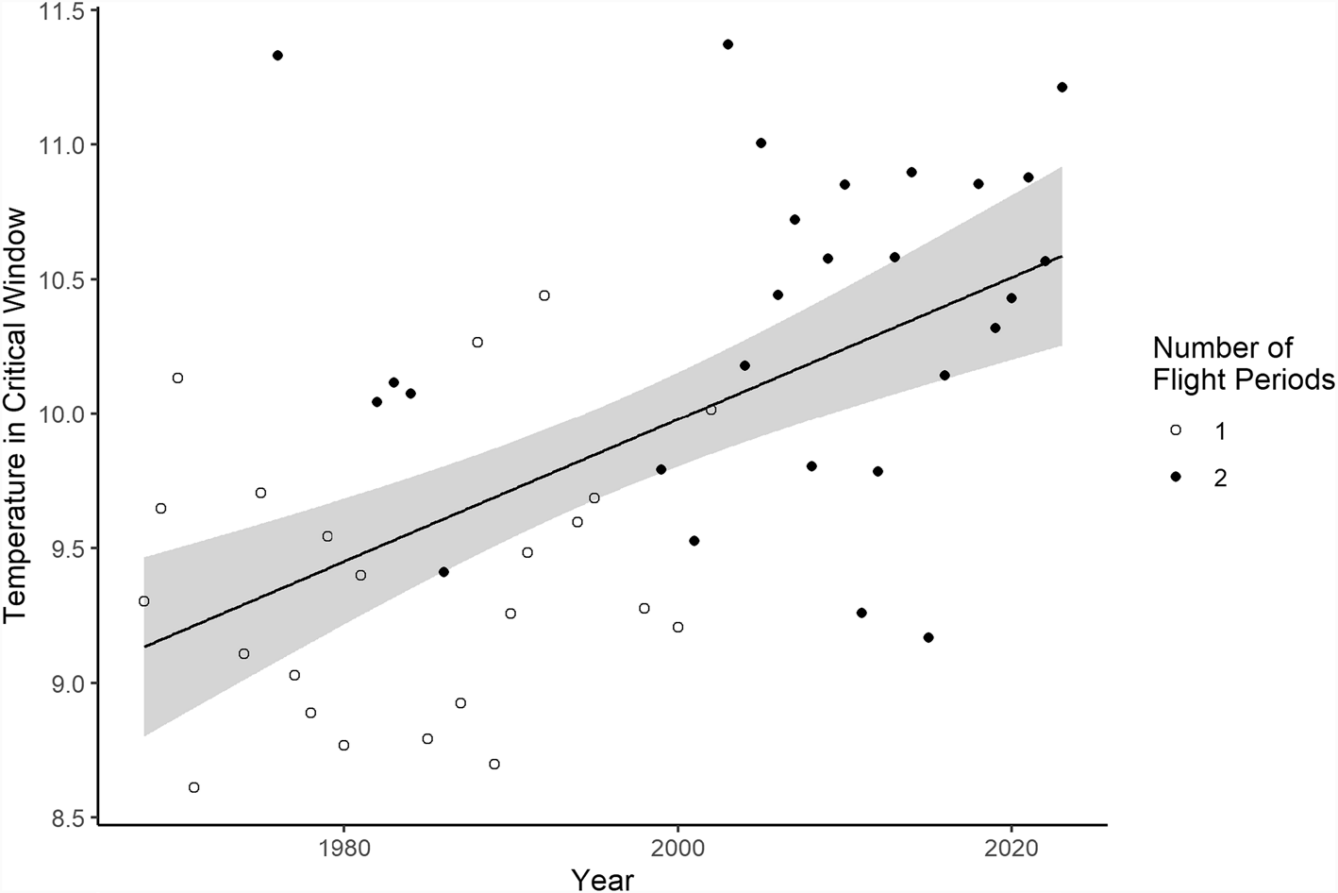
Plot of linear model of the mean daily minimum temperature between 1st June and 19th July on year. Points show observed values (*n* = 50) and are filled according to the number of flight periods observed in each year. Solid line shows fitted line from linear model. Shaded area shows the 95% confidence interval

Mean daily minimum temperature between 1st June and 19th July increased by 0.026 °C per year (t_1,54_ = 4.97, *p* < 0.001; Fig. 4). Using the binomial GLM of number of flight periods on mean daily minimum temperature in this critical window, the year at which the occurrence of a second flight period reached a probability of 0.5 (mean daily minimum temperature in critical time window = 10.0 °C) was 2000, which almost matches the observed data of consistent second flight periods from 2003 onwards.

Abundance during the first flight period in each year was best explained by abundance of the previous flight period (2ΔLL = 11.24, df = 1, *p* < 0.01) and by mean October to June temperature, which had a marginally significant effect (2ΔLL = 2.82, df = 1, *p* = 0.093). The model was not improved by including whether the previous flight period was a first or a second annual flight period (2ΔLL = 1.16, df = 1, *p* = 0.28). For every increase in the previous flight period’s abundance by 1 individual, first flight period abundance in each year increased by a factor of 1.03, whilst for each degree of increase in mean temperature between October and June, first flight period abundance increased by a factor of 1.30.

## Discussion

We found that the number of flight periods per year in this population of *E. silaceata* increased over time in accordance with hypothesis one, with the odds of a second flight period becoming 1.14 times greater each year. Furthermore, this increase in number of flight periods can be attributed to increased minimum temperature in a particular time window, with a 16.24-fold increase in the odds of a second flight period with every degree of temperature increase in this time window, and this effect is likely to facilitate population growth.

We found that the occurrence of a second flight period of *E. silaceata* can be explained by higher mean daily minimum temperatures between 1st June and 19th July. Critical windows chosen using more recent subsets of data had shorter window durations and smaller effect sizes of temperature on probability of a second flight period. This could be explained by the lower variation in the response variable (presence of a second flight period) in the more recent part of the data set, as further temperature increases beyond the point at which a second flight period emerges would be unlikely to have much additional effect on presence of a second flight period. The timing of the critical window did not change with subsample timing or duration, indicating that there was no change in the temperature cue used to initiate emergence of a second generation, or in other reaction norms which could alter this response (for example responses to photoperiod) throughout the data set. Furthermore, the high predictive performance shown by K-fold cross validation supports the validity of the approach used and suggests that the sliding window regression yielded a good estimate of the critical temperature window for presence of a second flight period in this population. Additionally, there was no bias in predictions generated from K-fold cross validation, providing further support that the cue driving variation in presence of a second generation was captured by this modelling approach and that there were not underlying drivers unexplained by the model. We also found that mean daily minimum temperature in the identified critical time window (1st June to 19th July) significantly increased throughout the 56 years of the study. These results provide support for hypothesis two by showing that occurrence of a second flight period can be explained by increased mean daily minimum temperature in a critical time window.

The critical time window (1st June to 19th July) begins approximately 1 month after the initial emergence of the first flight period (earliest date caught: 5th May (recorded in 2014 and 2022)), continuing until shortly before the first emergences of the second flight period (commencing beginning of August, according to Gaussian mixture model). This period is likely to occur when individuals in the first flight period of each year are laying eggs and when these eggs are developing into larvae and subsequently pupae. From the timings of the flight periods and knowledge of the natural history of this species, it is likely that the occurrence of the second flight period represents a second generation, and that the population studied here switched from univoltinism to bivoltinism. These results suggest that the direct transition into adults, and thus the occurrence of a second generation, is determined by environmental conditions during the development of that generation, rather than being predetermined by the emergence timing of their parent generation earlier in the year. *E. silaceata* overwinters as pupae, meaning that if it is to emerge as a second generation, this must be driven by the direct development of pupae into adults, and the consequent emergence of a second generation that year rather than overwintering in diapause. Therefore, the critical temperature window between the 1st of June and 19th of July, towards the later part of the adult emergence of the first generation, is likely to be particularly important during pupation of the second generation, driving progression into the adult stage.

Minimum temperature explained more variation in voltinism than mean or maximum temperature, suggesting that it is more important for development to the adult stage that temperatures remain above a certain minimum threshold rather than reaching a maximum threshold. The increase in mean daily minimum temperature in the identified critical window helps to explain the consistency of the occurrence of a second generation since 2003, as temperatures exceeded the threshold required for 0.5 probability of occurrence of a second generation (10.0 °C) in all but 4 years between 2003–2023. Similar increases in voltinism with increased environmental temperature over long time series have been observed in other ectotherms, including the communities of oak- (*Quercus spp.*) associated Lepidoptera in six countries across Europe (Gaytán et al. 2022).

There was a positive relationship between abundance of the first generation each year and abundance of the previous generation, regardless of the previous year’s voltinism, and when accounting for temperature. These results indicate positive density dependence in this population and support the idea that increased voltinism enables greater population growth by increasing the frequency at which positive density dependence can take effect, in accordance with hypothesis three. This contrasts with the idea that increased voltinism may have negative effects on abundance if later generations encounter less favourable environmental conditions. Bivoltinism in the pine bark beetle (*Ips acuminatus* (Gyllenhal, 1827)) in the Italian Alps appears to have negative consequences for population growth (Chinellato et al. 2014). Higher temperatures enable the first annual generation to reproduce prior to hibernation, leading to much larger second generations in warmer years. However, catch numbers decrease following warmer years, likely as temperatures later in the season are unsuitable for the second generation to complete their life cycle, resulting in high mortality of this later generation (Chinellato et al. 2014). The wall brown butterfly (*Lasiommata megera* (Linnaeus, 1767)) emerges in two or three generations per year across its range in northern Europe. This butterfly has shown substantial decreases in abundance, and a field experiment found that in areas where the butterfly is now extinct, introduced experimental animals consistently developed into a third annual generation, whereas in areas where the butterfly is still found naturally, less than half of individuals developed into a third generation. This suggests that the third generation encounters unfavourable conditions, leading to high mortality and population losses in areas where it occurs (Van Dyck et al. 2015). The results of our study suggest that this is not the case for the population studied here. Wepprich et al. (2025) found that, in 30 butterfly species in the USA, shifts to larger late-season generations have facilitated population growth. Although this study examined voltinism shifts (referring to changes in the relative abundance of generations within a year) rather than changes in voltinism (changes in the number of generations in a year), this study shows similar trends to the results found here for *E. silaceata*, with late-season flight periods enabling increased population growth (Wepprich et al. 2025). Based on our analysis, it appears likely that this population of *E. silaceata* will continue to increase in the immediate future, as there are no clear negative effects of bivoltinism, and the population does not appear to have reached a size sufficient for negative density dependence to occur, although this may take place after further population growth.

Although this species appears to have successfully increased voltinism in response to warming temperatures, this may not be possible for all species. Some species are obligately univoltine and are therefore unable to take advantage of the lengthened growing season with climate change, whilst other species may increase their number of generations to their detriment. In European Lepidoptera, the capacity for multivoltinism has been linked with host plant type, with reliance on woody plants constraining many species to univoltinism, whilst species feeding on herbaceous plants show more flexibility in voltinism (Cizek et al. 2006). Consequently, changes in voltinism could change the composition of the community of Lepidoptera with respect to their feeding, thereby altering their impacts as herbivores and pollinators. The larvae of *E. silaceata* feed primarily on willowherbs, as do the larvae of several other moth species recorded in the light trap at this site: setaceous Hebrew character (*Xestia c-nigrum* (Linnaeus, 1758)), the gothic (*Naenia typica* (Linnaeus, 1758)), small angle shades (*Euplexia lucipara* (Linnaeus, 1758)), twin-spot carpet (*Mesotype didymata* (Linnaeus, 1758)), elephant hawk-moth (*Deilephila elpenor* (Linnaeus, 1758)) and small elephant hawk-moth (*Deilephila porcellus* (Linnaeus, 1758)) (Waring & Townsend 2018). Changes in *E. silaceata* populations could alter competition with other herbivores for willowherbs, or if accompanied by changes in voltinism by other herbivores of willowherb, could alter the dynamics of herbivory in their ecosystem more broadly. Adult *E. silaceata* have a proboscis (pers. obs.) and therefore likely feed on nectar, potentially endowing them with a role as pollinators. Therefore, changes in the abundance of this species may have substantial consequences for the rest of the ecosystem, although those consequences are unclear given the lack of knowledge about the adult feeding behaviour of this species. Changes in voltinism and subsequent changes in population size and community composition at different times of year clearly have many potential consequences which remain to be investigated.

To confirm that increased voltinism is driven by higher minimum temperatures during the pupal stage, it would be valuable to conduct laboratory experiments rearing individuals under different temperature regimes to determine the responses of developmental processes to different temperature cues (Régnière et al. 2012). Experimental assessments of the effects of temperature on developmental rate throughout the life cycle could be used to construct a detailed model of the species’ phenology, as has been done for several pest species (Pollard et al. 2020; Tobin et al. 2001). Such approaches could be validated by comparing predicted voltinism based on environmental conditions to data observed in populations of *E. silaceata.* It would be particularly valuable to test developmental models in this way over multiple sites (as in Tobin et al. 2003), showing whether responses to key temperature variables differ between populations or across latitudinal gradients with differing photoperiod. Comparing phenological responses to environmental drivers across both time and space enables the mechanisms behind these responses (plasticity or adaptive evolution) to be identified (Phillimore et al. 2010, 2012), providing further information about the likely resilience of such responses under environmental change.

There are several other aspects of studying voltinism in *E. silaceata* which would benefit from research conducted across multiple sites. A shift from univoltinism to bivoltinism may indicate that the latitudinal boundary between bivoltine and univoltine life histories has shifted further north, previously occurring south of the site in west-central Scotland used in this study and now being found at higher latitude. To determine whether this is the case, further data gathered through light trapping, including at other sites in the Rothamsted Insect Survey, or observational data from amateur lepidopterists, could provide insight into the positioning of this latitudinal boundary and whether it has changed over time. Multivoltinism and phenological shifts in response to climate change have also been linked to range expansions in British Lepidoptera (Macgregor et al. 2019), which could be tested for *E. silaceata* using observations of the species at other sites across the UK.

In summary, we found that a population of *E. silaceata* in west-central Scotland had changed from univoltine to bivoltine over the period of 1968 to 2023. Increased voltinism in this population can be explained by increased minimum temperature in a critical time window towards the later part of the first generation’s flight period. This population shows positive density dependence, and increased voltinism has no significant negative effect on population size, indicating that increased voltinism here aids population growth. These results help to explain the causes of changes in voltinism, indicating the importance of sustained temperature above minimum thresholds for development. These results could also help to make predictions about future changes in population sizes under climate change and increasing voltinism, in addition to how these changes may differ between species, with community- and ecosystem-level effects. Further study of other populations and species under different environmental conditions in both field and lab studies is necessary to fully understand the effects of the environment on development and voltinism at individual and population levels.

## Data Availability

The data used in this study are available from the Rothamsted Insect Survey Online Database (RIS 2021) and the Met Office HadUK-Grid data set (Hollis et al. 2018). The data for this site (both the moth catch data and the temperature data) are available in the GitHub repository associated with this research (https://github.com/jguthrie2/voltinism).

## Code availability

The code used in this research is available in a GitHub repository at https://github.com/jguthrie2/voltinism

## References

[CR1] Altermatt F (2010) Climatic warming increases voltinism in European butterflies and moths. Proceed Roy Societ 277(1685):1281–1287. 10.1098/rspb.2009.1910 10.1098/rspb.2009.1910 PMC2842813 20031988

[CR2] Barnier J, Briatte F, & Larmarange J. (2025). *questionr: Functions to make surveys processing easier* (Version 0.8.1) [Computer software]. https://juba.github.io/questionr/

[CR3] Barton M, Sunnucks P, Norgate M, Murray N, Kearney M (2014) Co-gradient variation in growth rate and development time of a broadly distributed butterfly. PLoS ONE 9(4):e95258 24743771 10.1371/journal.pone.0095258 PMC3990641

[CR4] Belitz MW, Barve V, Doby JR, Hantak MM, Larsen EA, Li D, Oswald JA, Sewnath N, Walters M, Barve N, Earl K, Gardner N, Guralnick RP, Stucky BJ (2021) Climate drivers of adult insect activity are conditioned by life history traits. Ecol Lett 24(12):2687–2699. 10.1111/ele.13889 34636143 10.1111/ele.13889

[CR5] Benaglia T, Chauveau D, Hunter DR, Young D (2009) mixtools: An R package for analyzing finite mixture models. J Stat Softw 32(6):1–29

[CR6] Brooks ME, Kristensen K, van Benthem KJ, Magnusson A, Berg CW, Nielsen A, Skaug HJ, Maechler M, Bolker B (2017) glmmTMB balances speed and flexibility among packages for zero-inflated generalized linear mixed modeling. The R Journal 9(2):378–400

[CR7] Brown JH, Gillooly JF, Allen AP, Savage VM, West GB (2004) Toward a metabolic theory of ecology. Ecology 85(7):1771–1789

[CR8] Chen S, Fleischer SJ, Tobin PC, Saunders MC (2011) Projecting insect voltinism under high and low greenhouse gas emission conditions. Environ Entomol 40(3):505–515. 10.1603/EN10099 22251628 10.1603/EN10099

[CR9] Chinellato F, Battisti A, Finozzi V, Faccoli M (2014) Better today but worse tomorrow: how warm summers affect breeding performance of a Scots pine pest. Agrochimica 58(1):133–145

[CR10] Chinery M (2005) Collins complete guide to British insects. HarperCollins Publishers, France

[CR11] Choi WI, Park YK, Park YS, Ryoo MI, Lee HP (2011) Changes in voltinism in a pine moth Dendrolimus spectabilis (Lepidoptera: Lasiocampidae) population: Implications of climate change. Appl Entomol Zool 46(3):319–325. 10.1007/s13355-011-0046-x

[CR12] Cizek L, Fric Z, Konvicka M (2006) Host plant defences and voltinism in European butterflies. Ecol Entomol 31(4):337–344. 10.1111/j.1365-2311.2006.00783.x

[CR13] Dixon AFG, Honěk A, Keil P, Kotela MAA, Šizling AL, Jarošík V (2009) Relationship between the minimum and maximum temperature thresholds for development in insects. Funct Ecol 23(2):257–264. 10.1111/j.1365-2435.2008.01489.x

[CR14] Gaytán Á, Gotthard K, Tack AJM (2022) Strong impact of temperature and resource specialisation on patterns of voltinism within an oak-associated insect community. Ecol Entomol 47(4):544–552. 10.1111/een.13139

[CR15] Gotthard K, Nylin S, Wiklund C (2000) Individual state controls temperature dependence in a butterfly (Lasiommata maera). Proceed Royal Soc 267(1443):589–593. 10.1098/rspb.2000.1042 10.1098/rspb.2000.1042 PMC1690575 10787163

[CR16] Grevstad FS, Coop LB (2015) The consequences of photoperiodism for organisms in new climates. Ecol Appl 25(6):1506–1517. 10.1890/14-2071.1 26552260 10.1890/14-2071.1

[CR17] Hartig, F. (2022). *DHARMa: Residual diagnostics for hierarchical (multi-level/mixed) regression models. R package version 0.4.5*.

[CR18] Hollis D, McCarthy M, Kendon M, Legg T, Simpson I (2018) HadUK-Grid gridded and regional average climate observations for the UK. Centre Environ Data Analy. 10.1002/gdj3.78

[CR19] Kerr NZ, Wepprich T, Grevstad FS, Dopman EB, Chew FS, Crone EE (2019) Developmental trap or demographic bonanza? Opposing consequences of earlier phenology in a changing climate for a multivoltine butterfly. Glob Change Biol 26(4):2014–2027. 10.1111/gcb.14959 10.1111/gcb.14959 31833162

[CR20] Kogan M, Prokopy R (2009) Agricultural entomology. In: Resh VH, Cardé RT (eds) *Encyclopedia of Insects* (Second edition). Academic Press, France

[CR21] Kong JD, Hoffmann AA, Kearney MR (2019) Linking thermal adaptation and life-history theory explains latitudinal patterns of voltinism. Philosop Transact Royal Soc. 10.1098/rstb.2018.0547 10.1098/rstb.2018.0547 PMC6606465 31203762

[CR22] Koštál V (2006) Eco-physiological phases of insect diapause. J Insect Physiol 52(2):113–127. 10.1016/j.jinsphys.2005.09.008 16332347 10.1016/j.jinsphys.2005.09.008

[CR23] Macgregor CJ, Thomas CD, Roy DB, Beaumont MA, Bell JR, Brereton T, Bridle JR, Dytham C, Fox R, Gotthard K, Hoffmann AA, Martin G, Middlebrook I, Nylin S, Platts PJ, Rasteiro R, Saccheri IJ, Villoutreix R, Wheat CW, Hill JK (2019) Climate-induced phenology shifts linked to range expansions in species with multiple reproductive cycles per year. Nature Communicat. 10.1038/s41467-019-12479-w 10.1038/s41467-019-12479-w PMC6813360 31649267

[CR24] McGregor R (1997) Influence of photoperiod on larval development in the leafmining moth Phyllonorycter mespilella (Lepidoptera: Gracillaridae). Ann Entomol Soc Am 90(3):333–336. 10.1093/aesa/90.3.333

[CR25] Phillimore AB, Hadfield JD, Jones OR, Smithers RJ (2010) Differences in spawning date between populations of common frog reveal local adaptation. Proc Natl Acad Sci USA 107(18):8292–8297. 10.1073/pnas.0913792107 20404185 10.1073/pnas.0913792107 PMC2889515

[CR26] Phillimore AB, Stålhandske S, Smithers RJ, Bernard R (2012) Dissecting the contributions of plasticity and local adaptation to the phenology of a butterfly and its host plants. Am Nat 180(5):655–670. 10.1086/667893 23070325 10.1086/667893

[CR27] Pollard CP, Griffin CT, de Andrade Moral R, Duffy C, Chuche J, Gaffney MT, Fealy RM, Fealy R (2020) phenModel: a temperature-dependent phenology/voltinism model for a herbivorous insect incorporating facultative diapause and budburst. Ecol Model 416:108910. 10.1016/j.ecolmodel.2019.108910

[CR28] Pöyry J, Leinonen R, Söderman G, Nieminen M, Heikkinen RK, Carter TR (2011) Climate-induced increase of moth multivoltinism in boreal regions. Glob Ecol Biogeogr 20(2):289–298. 10.1111/j.1466-8238.2010.00597.x

[CR29] R Core Team. (2021). *R: A language and environment for statistical computing*. R Foundation for Statistical Computing.

[CR30] Régnière J, Powell J, Bentz B, Nealis V (2012) Effects of temperature on development, survival and reproduction of insects: Experimental design, data analysis and modeling. J Insect Physiol 58(5):634–647. 10.1016/j.jinsphys.2012.01.010 22310012 10.1016/j.jinsphys.2012.01.010

[CR31] RIS. (2021). *Rothamsted Insect Survey Online Database*. 10.23637/rothamsted.987q7

[CR32] Roberts AMI, Tansey C, Smithers RJ, Phillimore AB (2015) Predicting a change in the order of spring phenology in temperate forests. Glob Change Biol 21(7):2603–2611. 10.1111/gcb.12896 10.1111/gcb.12896 PMC4964954 25731862

[CR33] Simmonds EG, Cole EF, Sheldon BC (2019) Cue identification in phenology: a case study of the predictive performance of current statistical tools. J Anim Ecol 88(9):1428–1440. 10.1111/1365-2656.13038 31162635 10.1111/1365-2656.13038 PMC8629117

[CR34] Tobin PC, Nagarkatti S, Saunders MC (2001) Modeling development in grape berry moth (Lepidoptera: Tortricidae). Environ Entomol 30(4):692–699. 10.1603/0046-225X-30.4.692

[CR35] Tobin PC, Nagarkatti S, Saunders MC (2003) Phenology of grape berry moth (Lepidoptera: Tortricidae) in cultivated grape at selected geographic locations. Environ Entomol 32(2):340–346. 10.1603/0046-225X-32.2.340

[CR36] Ushey K (2018) RcppRoll: Efficient rolling/windowed operations. R package version 0.3.0

[CR37] Van Dyck H, Bonte D, Puls R, Gotthard K, Maes D (2015) The lost generation hypothesis: Could climate change drive ectotherms into a developmental trap? Oikos 124(1):54–61. 10.1111/oik.02066

[CR38] Waring P, Townsend M (2018) *Field guide to the moths of Great Britain and Ireland* (3rd edn). Bloomsbury Publishing

[CR39] Wepprich T, Henry E, Haddad NM (2025) Voltinism shifts in response to climate warming generally benefit populations of multivoltine butterflies. Ecol Lett 28(4):e70018. 10.1111/ele.70018 40172582 10.1111/ele.70018

[CR40] Wickham H (2016) ggplot2: Elegant graphics for data analysis. Springer-Verlag. 10.18637/jss.v077.b02

[CR41] Williams CB (1948) The Rothamsted light trap. Proceed Royal Entomol Societ London 23(7–9):80–85

[CR42] Zeileis A, Hothorn T (2002) Diagnostic checking in regression relationships. R News 2(3):7–10

[CR43] Zeileis A, Kleiber C, Jackman S (2008) Regression models for count data in R. J Statist Soft. 10.18637/jss.v027.i08

